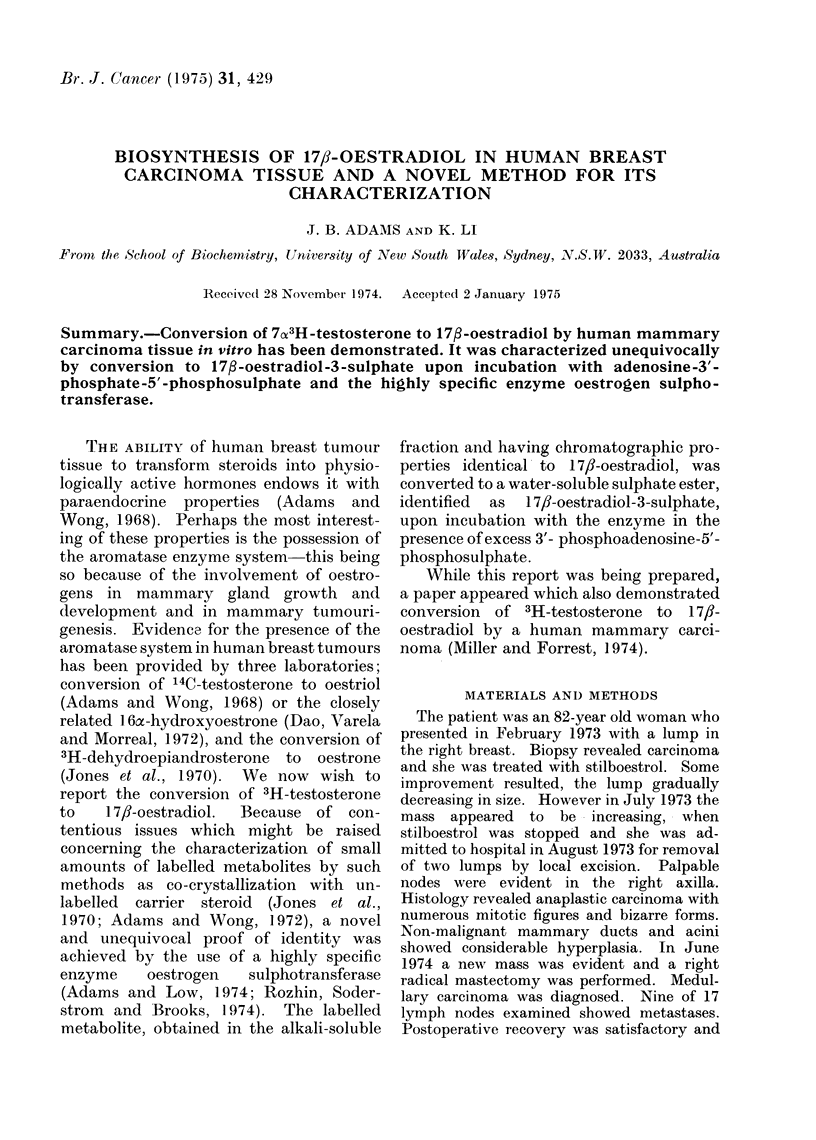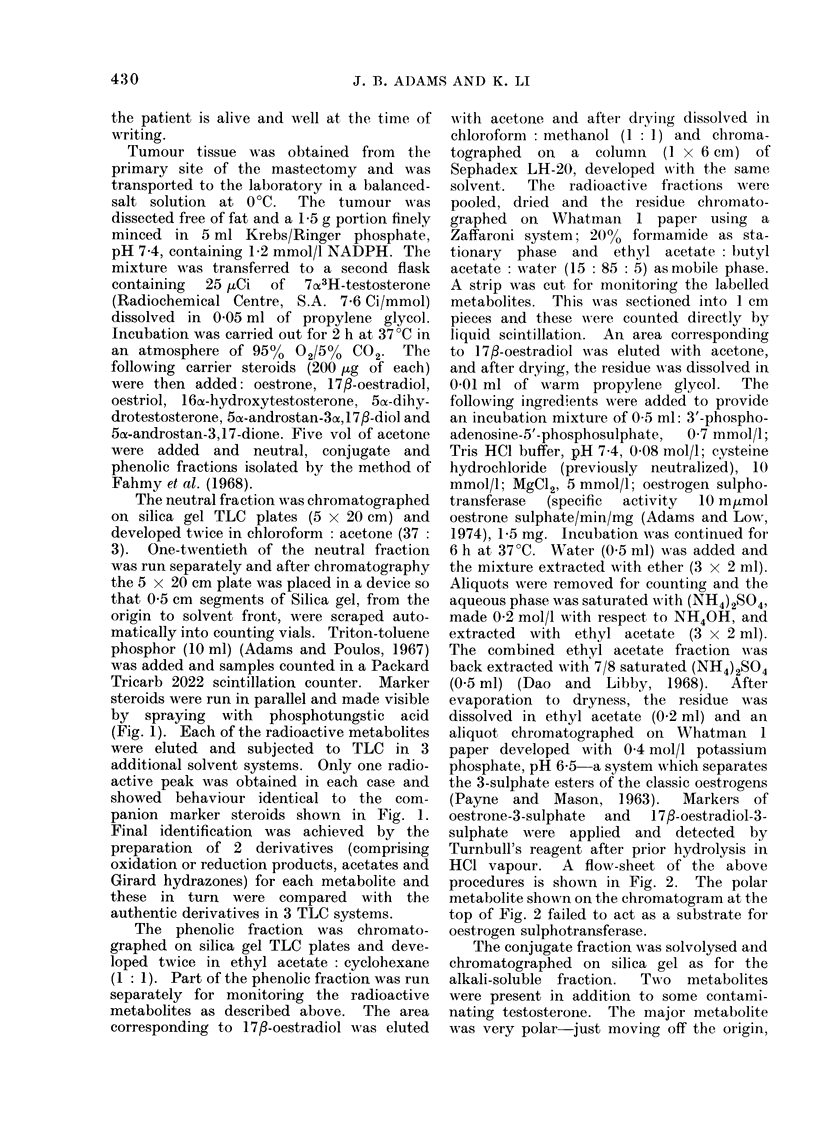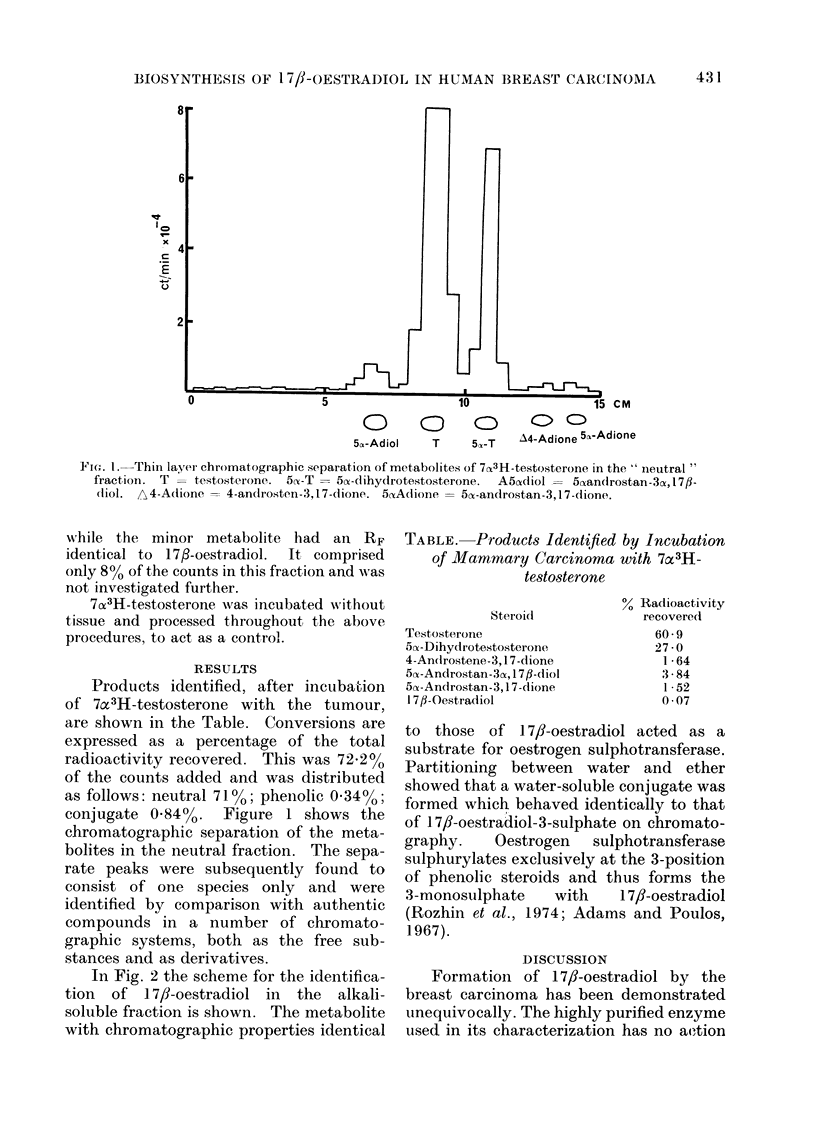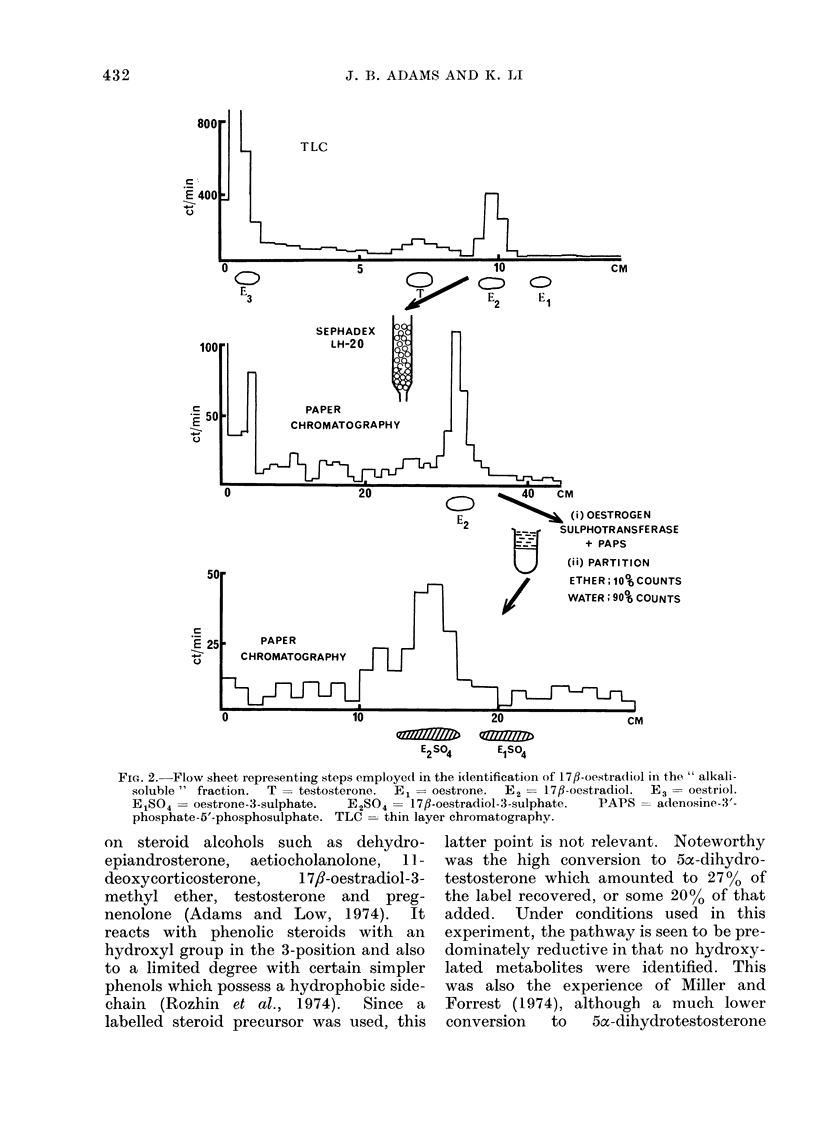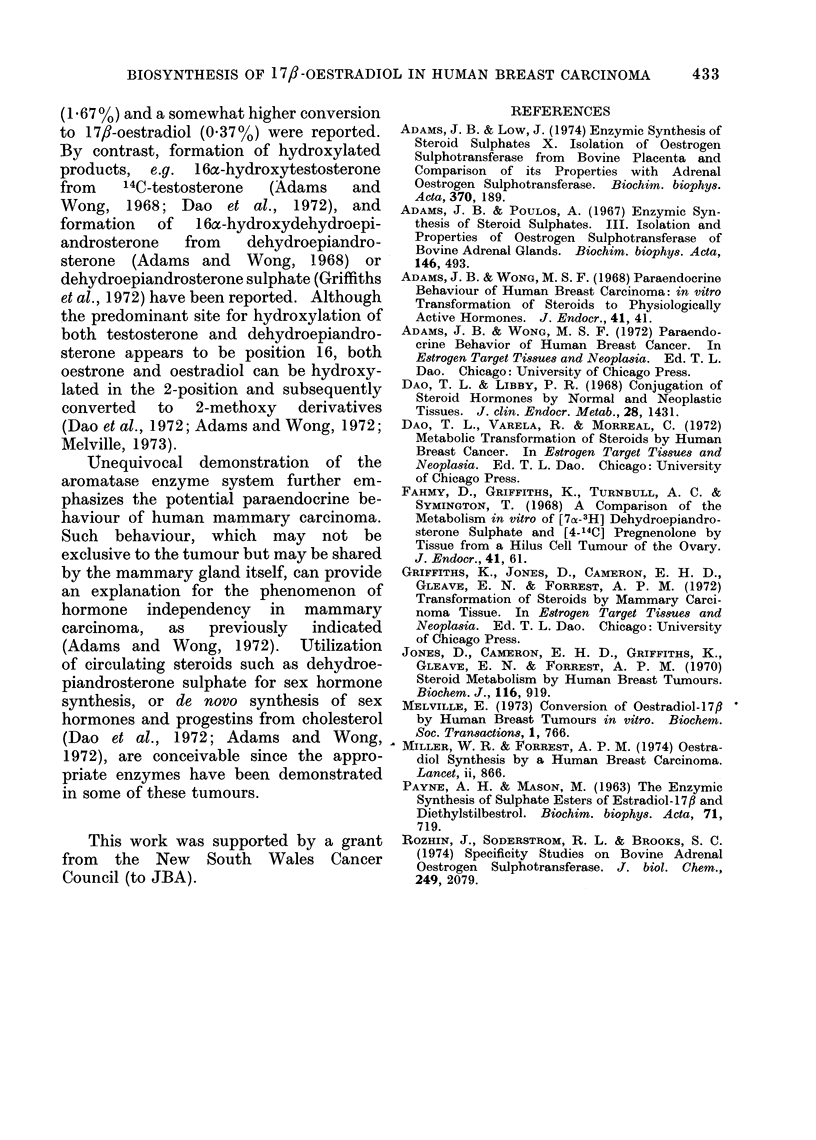# Biosynthesis of 17beta-oestradiol in human breast carcinoma tissue and a novel method for its characterization.

**DOI:** 10.1038/bjc.1975.82

**Published:** 1975-04

**Authors:** J. B. Adams, K. Li

## Abstract

Conversion of 7alpha3H-testosterone to 17beta-oestradiol by human mammary carcinoma tissue in vitro has been demonstrated. It was characterized unequivocally by conversion to 17beta-oestradiol-3-sulphate upon incubation with adenosine-3'-phosphate-5'-phosphosulphate and the highly specific enzyme oestrogen sulphotransferase.


*Br. J. Cancer* (1975) **31**, 429

### BIOSYNTHESIS OF 17β-OESTRADIOL IN HUMAN BREAST CARCINOMA TISSUE AND A NOVEL METHOD FOR ITS CHARACTERIZATION

J. B. ADAMS AND K. LI

*From the School of Biochemistry, University of New South Wales, Sydney, N.S.W. 2033, Australia*

Received 28 November 1974. Accepted 2 January 1975

**Summary.—Conversion of 7α$^3$H-testosterone to 17β-oestradiol by human mammary carcinoma tissue *in vitro* has been demonstrated. It was characterized unequivocally by conversion to 17β-oestradiol-3-sulphate upon incubation with adenosine-3′-phosphate-5′-phosphosulphate and the highly specific enzyme oestrogen sulphotransferase.**

THE ABILITY of human breast tumour tissue to transform steroids into physiologically active hormones endows it with paraendocrine properties (Adams and Wong, 1968). Perhaps the most interesting of these properties is the possession of the aromatase enzyme system—this being so because of the involvement of oestrogens in mammary gland growth and development and in mammary tumourigenesis. Evidence for the presence of the aromatase system in human breast tumours has been provided by three laboratories; conversion of $^{14}$C-testosterone to oestriol (Adams and Wong, 1968) or the closely related 16α-hydroxyoestrone (Dao, Varela and Morreal, 1972), and the conversion of $^3$H-dehydroepiandrosterone to oestrone (Jones *et al.*, 1970). We now wish to report the conversion of $^3$H-testosterone to 17β-oestradiol. Because of contentious issues which might be raised concerning the characterization of small amounts of labelled metabolites by such methods as co-crystallization with unlabelled carrier steroid (Jones *et al.*, 1970; Adams and Wong, 1972), a novel and unequivocal proof of identity was achieved by the use of a highly specific enzyme oestrogen sulphotransferase (Adams and Low, 1974; Rozhin, Soderstrom and Brooks, 1974). The labelled metabolite, obtained in the alkali-soluble fraction and having chromatographic properties identical to 17β-oestradiol, was converted to a water-soluble sulphate ester, identified as 17β-oestradiol-3-sulphate, upon incubation with the enzyme in the presence of excess 3′- phosphoadenosine-5′-phosphosulphate.

While this report was being prepared, a paper appeared which also demonstrated conversion of $^3$H-testosterone to 17β-oestradiol by a human mammary carcinoma (Miller and Forrest, 1974).

### MATERIALS AND METHODS

The patient was an 82-year old woman who presented in February 1973 with a lump in the right breast. Biopsy revealed carcinoma and she was treated with stilboestrol. Some improvement resulted, the lump gradually decreasing in size. However in July 1973 the mass appeared to be increasing, when stilboestrol was stopped and she was admitted to hospital in August 1973 for removal of two lumps by local excision. Palpable nodes were evident in the right axilla. Histology revealed anaplastic carcinoma with numerous mitotic figures and bizarre forms. Non-malignant mammary ducts and acini showed considerable hyperplasia. In June 1974 a new mass was evident and a right radical mastectomy was performed. Medullary carcinoma was diagnosed. Nine of 17 lymph nodes examined showed metastases. Postoperative recovery was satisfactory and the patient is alive and well at the time of writing.

Tumour tissue was obtained from the primary site of the mastectomy and was transported to the laboratory in a balanced-salt solution at 0°C. The tumour was dissected free of fat and a 1·5 g portion finely minced in 5 ml Krebs/Ringer phosphate, pH 7·4, containing 1·2 mmol/l NADPH. The mixture was transferred to a second flask containing 25 $\mu$Ci of 7$\alpha$$^3$H-testosterone (Radiochemical Centre, S.A. 7·6 Ci/mmol) dissolved in 0·05 ml of propylene glycol. Incubation was carried out for 2 h at 37°C in an atmosphere of 95% $O_2$/5% $CO_2$. The following carrier steroids (200 $\mu$g of each) were then added: oestrone, 17$\beta$-oestradiol, oestriol, 16$\alpha$-hydroxytestosterone, 5$\alpha$-dihydrotestosterone, 5$\alpha$-androstan-3$\alpha$,17$\beta$-diol and 5$\alpha$-androstan-3,17-dione. Five vol of acetone were added and neutral, conjugate and phenolic fractions isolated by the method of Fahmy *et al.* (1968).

The neutral fraction was chromatographed on silica gel TLC plates (5 × 20 cm) and developed twice in chloroform : acetone (37 : 3). One-twentieth of the neutral fraction was run separately and after chromatography the 5 × 20 cm plate was placed in a device so that 0·5 cm segments of Silica gel, from the origin to solvent front, were scraped automatically into counting vials. Triton-toluene phosphor (10 ml) (Adams and Poulos, 1967) was added and samples counted in a Packard Tricarb 2022 scintillation counter. Marker steroids were run in parallel and made visible by spraying with phosphotungstic acid (Fig. 1). Each of the radioactive metabolites were eluted and subjected to TLC in 3 additional solvent systems. Only one radioactive peak was obtained in each case and showed behaviour identical to the companion marker steroids shown in Fig. 1. Final identification was achieved by the preparation of 2 derivatives (comprising oxidation or reduction products, acetates and Girard hydrazones) for each metabolite and these in turn were compared with the authentic derivatives in 3 TLC systems.

The phenolic fraction was chromatographed on silica gel TLC plates and developed twice in ethyl acetate : cyclohexane (1 : 1). Part of the phenolic fraction was run separately for monitoring the radioactive metabolites as described above. The area corresponding to 17$\beta$-oestradiol was eluted with acetone and after drying dissolved in chloroform : methanol (1 : 1) and chromatographed on a column (1 × 6 cm) of Sephadex LH-20, developed with the same solvent. The radioactive fractions were pooled, dried and the residue chromatographed on Whatman 1 paper using a Zaffaroni system; 20% formamide as stationary phase and ethyl acetate : butyl acetate : water (15 : 85 : 5) as mobile phase. A strip was cut for monitoring the labelled metabolites. This was sectioned into 1 cm pieces and these were counted directly by liquid scintillation. An area corresponding to 17$\beta$-oestradiol was eluted with acetone, and after drying, the residue was dissolved in 0·01 ml of warm propylene glycol. The following ingredients were added to provide an incubation mixture of 0·5 ml: 3′-phosphoadenosine-5′-phosphosulphate, 0·7 mmol/l; Tris HCl buffer, pH 7·4, 0·08 mol/l; cysteine hydrochloride (previously neutralized), 10 mmol/l; $MgCl_2$, 5 mmol/l; oestrogen sulphotransferase (specific activity 10 m$\mu$mol oestrone sulphate/min/mg (Adams and Low, 1974), 1·5 mg. Incubation was continued for 6 h at 37°C. Water (0·5 ml) was added and the mixture extracted with ether (3 × 2 ml). Aliquots were removed for counting and the aqueous phase was saturated with $(NH_4)_2SO_4$, made 0·2 mol/l with respect to $NH_4OH$, and extracted with ethyl acetate (3 × 2 ml). The combined ethyl acetate fraction was back extracted with 7/8 saturated $(NH_4)_2SO_4$ (0·5 ml) (Dao and Libby, 1968). After evaporation to dryness, the residue was dissolved in ethyl acetate (0·2 ml) and an aliquot chromatographed on Whatman 1 paper developed with 0·4 mol/l potassium phosphate, pH 6·5—a system which separates the 3-sulphate esters of the classic oestrogens (Payne and Mason, 1963). Markers of oestrone-3-sulphate and 17$\beta$-oestradiol-3-sulphate were applied and detected by Turnbull's reagent after prior hydrolysis in HCl vapour. A flow-sheet of the above procedures is shown in Fig. 2. The polar metabolite shown on the chromatogram at the top of Fig. 2 failed to act as a substrate for oestrogen sulphotransferase.

The conjugate fraction was solvolysed and chromatographed on silica gel as for the alkali-soluble fraction. Two metabolites were present in addition to some contaminating testosterone. The major metabolite was very polar—just moving off the origin,

FIG. 1.—Thin layer chromatographic separation of metabolites of 7α³H-testosterone in the " neutral " fraction. T = testosterone. 5α-T = 5α-dihydrotestosterone. A5αdiol = 5αandrostan-3α,17β-diol. △4-Adione = 4-androsten-3,17-dione. 5αAdione = 5α-androstan-3,17-dione.

while the minor metabolite had an $R_F$ identical to 17β-oestradiol. It comprised only 8% of the counts in this fraction and was not investigated further.

7α³H-testosterone was incubated without tissue and processed throughout the above procedures, to act as a control.

### RESULTS

Products identified, after incubation of 7α³H-testosterone with the tumour, are shown in the Table. Conversions are expressed as a percentage of the total radioactivity recovered. This was 72·2% of the counts added and was distributed as follows: neutral 71%; phenolic 0·34%; conjugate 0·84%. Figure 1 shows the chromatographic separation of the metabolites in the neutral fraction. The separate peaks were subsequently found to consist of one species only and were identified by comparison with authentic compounds in a number of chromatographic systems, both as the free substances and as derivatives.

In Fig. 2 the scheme for the identification of 17β-oestradiol in the alkali-soluble fraction is shown. The metabolite with chromatographic properties identical to those of 17β-oestradiol acted as a substrate for oestrogen sulphotransferase. Partitioning between water and ether showed that a water-soluble conjugate was formed which behaved identically to that of 17β-oestradiol-3-sulphate on chromatography. Oestrogen sulphotransferase sulphurylates exclusively at the 3-position of phenolic steroids and thus forms the 3-monosulphate with 17β-oestradiol (Rozhin *et al.*, 1974; Adams and Poulos, 1967).

TABLE.—*Products Identified by Incubation of Mammary Carcinoma with 7α³H-testosterone*

| Steroid | % Radioactivity recovered |
|---|---|
| Testosterone | 60·9 |
| 5α-Dihydrotestosterone | 27·0 |
| 4-Androstene-3,17-dione | 1·64 |
| 5α-Androstan-3α,17β-diol | 3·84 |
| 5α-Androstan-3,17-dione | 1·52 |
| 17β-Oestradiol | 0·07 |

### DISCUSSION

Formation of 17β-oestradiol by the breast carcinoma has been demonstrated unequivocally. The highly purified enzyme used in its characterization has no action

FIG. 2.—Flow sheet representing steps employed in the identification of 17$\beta$-oestradiol in the " alkali-soluble " fraction. T = testosterone. $E_1$ = oestrone. $E_2$ = 17$\beta$-oestradiol. $E_3$ = oestriol. $E_1SO_4$ = oestrone-3-sulphate. $E_2SO_4$ = 17$\beta$-oestradiol-3-sulphate. PAPS = adenosine-3'-phosphate-5'-phosphosulphate. TLC = thin layer chromatography.

on steroid alcohols such as dehydroepiandrosterone, aetiocholanolone, 11-deoxycorticosterone, 17$\beta$-oestradiol-3-methyl ether, testosterone and pregnenolone (Adams and Low, 1974). It reacts with phenolic steroids with an hydroxyl group in the 3-position and also to a limited degree with certain simpler phenols which possess a hydrophobic side-chain (Rozhin *et al.*, 1974). Since a labelled steroid precursor was used, this latter point is not relevant. Noteworthy was the high conversion to 5$\alpha$-dihydrotestosterone which amounted to 27% of the label recovered, or some 20% of that added. Under conditions used in this experiment, the pathway is seen to be predominately reductive in that no hydroxylated metabolites were identified. This was also the experience of Miller and Forrest (1974), although a much lower conversion to 5$\alpha$-dihydrotestosterone

(1·67%) and a somewhat higher conversion to 17$\beta$-oestradiol (0·37%) were reported. By contrast, formation of hydroxylated products, *e.g.* 16$\alpha$-hydroxytestosterone from $^{14}$C-testosterone (Adams and Wong, 1968; Dao *et al.*, 1972), and formation of 16$\alpha$-hydroxydehydroepiandrosterone from dehydroepiandrosterone (Adams and Wong, 1968) or dehydroepiandrosterone sulphate (Griffiths *et al.*, 1972) have been reported. Although the predominant site for hydroxylation of both testosterone and dehydroepiandrosterone appears to be position 16, both oestrone and oestradiol can be hydroxylated in the 2-position and subsequently converted to 2-methoxy derivatives (Dao *et al.*, 1972; Adams and Wong, 1972; Melville, 1973).

Unequivocal demonstration of the aromatase enzyme system further emphasizes the potential paraendocrine behaviour of human mammary carcinoma. Such behaviour, which may not be exclusive to the tumour but may be shared by the mammary gland itself, can provide an explanation for the phenomenon of hormone independency in mammary carcinoma, as previously indicated (Adams and Wong, 1972). Utilization of circulating steroids such as dehydroepiandrosterone sulphate for sex hormone synthesis, or *de novo* synthesis of sex hormones and progestins from cholesterol (Dao *et al.*, 1972; Adams and Wong, 1972), are conceivable since the appropriate enzymes have been demonstrated in some of these tumours.

This work was supported by a grant from the New South Wales Cancer Council (to JBA).

REFERENCES

Adams, J. B. & Low, J. (1974) Enzymic Synthesis of Steroid Sulphates X. Isolation of Oestrogen Sulphotransferase from Bovine Placenta and Comparison of its Properties with Adrenal Oestrogen Sulphotransferase. *Biochim. biophys. Acta*, **370**, 189.

Adams, J. B. & Poulos, A. (1967) Enzymic Synthesis of Steroid Sulphates. III. Isolation and Properties of Oestrogen Sulphotransferase of Bovine Adrenal Glands. *Biochim. biophys. Acta*, **146**, 493.

Adams, J. B. & Wong, M. S. F. (1968) Paraendocrine Behaviour of Human Breast Carcinoma: *in vitro* Transformation of Steroids to Physiologically Active Hormones. *J. Endocr.*, **41**, 41.

Adams, J. B. & Wong, M. S. F. (1972) Paraendocrine Behavior of Human Breast Cancer. In *Estrogen Target Tissues and Neoplasia.* Ed. T. L. Dao. Chicago: University of Chicago Press.

Dao, T. L. & Libby, P. R. (1968) Conjugation of Steroid Hormones by Normal and Neoplastic Tissues. *J. clin. Endocr. Metab.*, **28**, 1431.

Dao, T. L., Varela, R. & Morreal, C. (1972) Metabolic Transformation of Steroids by Human Breast Cancer. In *Estrogen Target Tissues and Neoplasia.* Ed. T. L. Dao. Chicago: University of Chicago Press.

Fahmy, D., Griffiths, K., Turnbull, A. C. & Symington, T. (1968) A Comparison of the Metabolism *in vitro* of [7$\alpha$-$^3$H] Dehydroepiandrosterone Sulphate and [4-$^{14}$C] Pregnenolone by Tissue from a Hilus Cell Tumour of the Ovary. *J. Endocr.*, **41**, 61.

Griffiths, K., Jones, D., Cameron, E. H. D., Gleave, E. N. & Forrest, A. P. M. (1972) Transformation of Steroids by Mammary Carcinoma Tissue. In *Estrogen Target Tissues and Neoplasia.* Ed. T. L. Dao. Chicago: University of Chicago Press.

Jones, D., Cameron, E. H. D., Griffiths, K., Gleave, E. N. & Forrest, A. P. M. (1970) Steroid Metabolism by Human Breast Tumours. *Biochem. J.*, **116**, 919.

Melville, E. (1973) Conversion of Oestradiol-17$\beta$ by Human Breast Tumours *in vitro*. *Biochem. Soc. Transactions*, **1**, 766.

Miller, W. R. & Forrest, A. P. M. (1974) Oestradiol Synthesis by a Human Breast Carcinoma. *Lancet*, ii, 866.

Payne, A. H. & Mason, M. (1963) The Enzymic Synthesis of Sulphate Esters of Estradiol-17$\beta$ and Diethylstilbestrol. *Biochim. biophys. Acta*, **71**, 719.

Rozhin, J., Soderstrom, R. L. & Brooks, S. C. (1974) Specificity Studies on Bovine Adrenal Oestrogen Sulphotransferase. *J. biol. Chem.*, **249**, 2079.